# Pathway-based signatures predict patient outcome, chemotherapy benefit and synthetic lethal dependencies in invasive lobular breast cancer

**DOI:** 10.1038/s41416-024-02679-7

**Published:** 2024-04-10

**Authors:** John Alexander, Koen Schipper, Sarah Nash, Rachel Brough, Harriet Kemp, Jacopo Iacovacci, Clare Isacke, Rachael Natrajan, Elinor Sawyer, Christopher J. Lord, Syed Haider

**Affiliations:** 1 The Breast Cancer Now Toby Robins Research Centre, The Institute of Cancer Research, London, SW3 6JB UK; 2 Breast Cancer Genetics, King’s College London, London, SE1 9RT UK; 3 The CRUK Gene Function Laboratory, The Institute of Cancer Research, London, SW3 6JB UK

**Keywords:** Predictive markers, Breast cancer, Prognostic markers, Machine learning

## Abstract

**Background:**

Invasive Lobular Carcinoma (ILC) is a morphologically distinct breast cancer subtype that represents up to 15% of all breast cancers. Compared to Invasive Breast Carcinoma of No Special Type (IBC-NST), ILCs exhibit poorer long-term outcome and a unique pattern of metastasis. Despite these differences, the systematic discovery of robust prognostic biomarkers and therapeutically actionable molecular pathways in ILC remains limited.

**Methods:**

Pathway-centric multivariable models using statistical machine learning were developed and tested in seven retrospective clinico-genomic cohorts (*n* = 996). Further external validation was performed using a new RNA-Seq clinical cohort of aggressive ILCs (*n* = 48).

**Results and conclusions:**

mRNA dysregulation scores of 25 pathways were strongly prognostic in ILC (FDR-adjusted *P* < 0.05). Of these, three pathways including Cell-cell communication, Innate immune system and Smooth muscle contraction were also independent predictors of chemotherapy response. To aggregate these findings, a multivariable machine learning predictor called PSILC was developed and successfully validated for predicting overall and metastasis-free survival in ILC. Integration of PSILC with CRISPR-Cas9 screening data from breast cancer cell lines revealed 16 candidate therapeutic targets that were synthetic lethal with high-risk ILCs. This study provides interpretable prognostic and predictive biomarkers of ILC which could serve as the starting points for targeted drug discovery for this disease.

## Introduction

Invasive Lobular Carcinoma (ILC) is the second most frequently occurring histological subtype of breast cancer after Invasive Breast Carcinoma of No Special Type (IBC-NST, formerly referred to as invasive ductal carcinoma or IDC), representing up to 15% of all breast cancer cases (reviewed in [1–3]). ILC is commonly distinguished by small discohesive cancer cells permeating the stroma in an individually dispersed or single-file pattern [1, 2, 4]. The loss of the calcium-dependent cell-cell adhesion protein, E-cadherin (encoded by *CDH1*) is thought to cause the lack of cellular cohesion in ILCs and is seen in over 85% of ILCs [1, 5]. In addition to E-cadherin deficiency, ILCs exhibit a low proliferation index (Ki67), oestrogen receptor (ER) and progesterone receptor (PgR) positivity, and low tumour purity [2, 5]. Compared to ER positive (ER+) IBC-NST, ER+ ILCs present distinctive morphological and pathologic features with worse disease free and overall survival at 10 years, and unusual metastatic patterns [2, 6–8].

Large-scale genomic studies have revealed a higher alteration rate in driver genes *PIK3CA, TBX3, FOXA1*, *RUNX1* and *PTEN*, and a lower alteration rate in *TP53, MYC, ERBB2/3* and *GATA3* [5, 9, 10] in ILCs when compared to IBC-NST. Mutations in *PIK3CA* and *AKT1* along with *PTEN* inactivating events in ILCs lead to increased activity of the PI3K/Akt signalling pathway [5]. Pathways including MAPK and metabotropic glutamate receptor signalling [5], WNT4 signalling [11] and ERRγ/AP1 signalling [12] have also been linked to therapy resistance, suggesting ILCs are likely to be driven by a complex interplay between multiple molecular pathways.

Despite clear histological, molecular and clinical differences between ILCs and IBC-NST, the treatment options for the majority of ILCs (i.e. ER+) and IBC-NST remain the same, involving a combination of surgery, radiotherapy, chemotherapy and hormone therapy [13]. These treatment options are typically guided by immunohistochemical quantification of ER, PR, and HER2, and additional tests such as PREDICT [14] and Oncotype DX [15], neither of which takes morphology into account. Whilst current therapeutic options offer good short-term prognosis, longer-term outcome (beyond 5 years) of ILCs remains inferior, with ILCs displaying preferential metastatic propensity to bone and gastrointestinal tract compared to patients with the more commonly diagnosed IBC-NST [6, 7, 16]. There is, therefore, an unmet need to design molecular tests specifically for ILCs, that not only predict patient outcome and response to current therapies, but also reveal key underlying biology for the development of next-generation targeted therapies.

A number of existing molecular biomarkers originally designed for ER+ breast cancer subtypes have been tested in ILC cohorts with the aim of providing prognostic information beyond classic clinico-pathologic characteristics (reviewed in [2, 10, 17]). Although these biomarkers have shown prognostic value in ILC [18–22], they were neither developed to capture ILC-specific biology nor to predict late recurrences, which remains a key clinical challenge. Recent genome profiling efforts have led to the discovery of molecular subtypes of ILC [5, 23] as well as the first ILC risk predictor, LobSig [9]. Whilst these subtypes advance our understanding of the molecular underpinnings of ILCs, they lack independent validation across multiple datasets. Furthermore, the predictive potential of these ILC signatures, including their response to chemotherapy and derivation of novel ILC-specific drug targets, remains to be elucidated.

Here we developed prognostic biomarkers of lobular breast cancer using a robust pathway-centric approach [24–26] that enabled integration of ILC-specific biology. We tested these biomarkers retrospectively in a large series of six independent ILC cohorts and evaluated their potential in predicting response to chemotherapy in a contemporary clinical cohort (SCAN-B) [27]. Further, we developed and successfully validated a multivariable prognostic classifier that also revealed context-specific candidate synthetic lethal targets of aggressive ILCs.

## Methods

### Pre-processing of mRNA abundance profiles and metadata

Metabric: the Metabric breast cancer dataset was pre-processed, summarised and quantile-normalised from raw expression files generated by Illumina Bead-Studio using R packages beadarray v2.4.2 and illuminaHuman v3.db_1.12.2. Raw Metabric files were downloaded from the European genome-phenome archive (EGA) (Study ID: EGAS00000000083). log_2_ transformed probe level data was mapped to genes. Only the most variable probe was kept where multiple probes per gene were available.

TCGA (BRCA): TCGA clinical and RNA-Seq RSEM normalised data were downloaded from http://gdac.broadinstitute.org/ (Illumina HiSeq rnaseqv2 level 3 RSEM; release 2016-01-28). mRNA data was log_2_ transformed after adding a prior of 1. Genes where >75% of samples had zero counts were removed from the dataset.

RATHER: the dataset was downloaded from the GEO website using accession id GSE68057 along with its custom CDF (GPL20078). log_2_ normalised data for each probe was extracted and stored as an expression dataset. Probes were mapped to HGNC gene symbols using the provided custom CDF and were further mapped to EntrezIDs using the R package org.Hs.eg.db (v3.7.0). Where genes with multiple probes were present, we kept the most variable probe. Twenty-two lobular samples overlapping with our Metabric discovery cohort were removed from the RATHER validation dataset. We excluded lobular mixed non-classical and unspecified histology type samples from our analysis.

Metzger-Filho: the dataset and its metadata were downloaded from GEO using accession id GSE88770. ProbeSet annotation to Entrez IDs was done using the R package hgu133plus2hsentrezgcdf (v18.0.0). Data were normalised and log_2_ transformed using the R package affy (v1.60.0) with *justRMA()*.

Guedj: the dataset and its metadata were downloaded using the ArrayExpress (v1.42.0) R package using accession id E-MTAB-365. ProbeSet annotation to Entrez IDs was done using the R package hgu133plus2hsentrezgcdf (v18.0.0). Data were normalised and log_2_ transformed using the R package affy (v1.60.0) with *justRMA()*.

Sabatier: the dataset and its metadata were downloaded from GEO using accession id GSE21653. Pre-processing was performed as previously described here [26].

SCAN-B: normalised RNA-Seq data was downloaded from GEO using accession id GSE96058. We further removed repeat samples, exponentiated the data, removed the prior of 0.1, added a prior of 1 and subsequently log_2_ transformed the data. HGNC gene symbols were mapped to EntrezIDs using the R package org.Hs.eg.db (v3.7.0). Histology information was obtained from http://oncogenomics.bmc.lu.se/MutationExplorer ([28]). Genes where >75% of samples had zero counts were removed from the dataset.

CCLE: normalised RNA-Seq data was downloaded from the Cancer Cell Line Encyclopedia (CCLE) cell line portal https://sites.broadinstitute.org/ccle/, further limiting to breast cancer cell lines (*n* = 57). mRNA data were log_2_ transformed after adding a prior of 1. Cell lines were designated as “ILC-like” using previously published breast cancer cell line annotations by Michaut et al. [23] (of these, eight were present in CCLE), combined with an additional 15 breast cancer cell lines which had *CDH1* expression less than the median *CDH1* expression of the eight bona-fide “ILC-like” cell lines.

Overall survival (OS) was used as the survival end point, except for Guedj (metastasis-free survival) and Sabatier (disease free survival) where OS was not available. The analysis was limited to ILC histology only, excluding IBC-NST as well as mixed ILC/IBC-NST cases. Median follow-up time was estimated using the reverse Kaplan–Meier method [29].

### RNA-seq profiling and quantification of KCL cohort

RNA extraction for primary untreated ILC tumours (*n* = 50) was performed at the Innovation Hub, Guys Cancer Centre, King’s College London. RNA sequencing was performed at The Genomics Facility, The Institute of Cancer Research, Sutton. Tissue macro needle dissection was used to enrich for tumour content (minimum 50% cellularity) and 10 × 10 μm FFPE tissue sections were used per case. RNA was extracted using the Qiagen AllPrep DNA/RNA FFPE Tissue Kit (Qiagen, Manchester, UK). The quantity of extracted RNA was analysed using the Qubit Fluorometer (Fisher Scientific, Loughborough, UK). RNA extraction and next-generation sequencing (NGS) were completed at Good Clinical Laboratory Practice (GCLP)-accredited laboratories. Two hundred and fifty to 1000 ng of total RNA, from FFPE material, was treated with TurboDNase (Invitrogen, #AM2239) to remove genomic DNA contamination. Ribosomal RNA was then removed from the sample using the NEBNext rRNA Depletion Kit (NEB, #E6310X) following the manufacturer’s directions. From the resulting RNA, strand-specific libraries were created using the NEBNext Ultra II Directional RNA Library Prep Kit for Illumina (NEB, #E7760) on the Agilent Bravo (option B). Final libraries were quantified using TapeStation (Agilent) and qPCR (Roche, #KK4835), then clustered at a Molarity of 300 pM. Sequencing was performed on an Illumina NovaSeq 6000 using PE x100 cycles v1.5 chemistry, to achieve coverage of 100 million reads per sample. RNA-Seq profiling generated 25.6 to 143.8 million paired-end reads per sample. Library quality was assessed using FastQC, FastQ Screen (PMID: 30254741) and MultiQC (v1.9) (PMID: 27312411). Reads were trimmed using Trim Galore (v0.6.6). Paired-end reads were aligned to the human reference genome GRCh38, using STAR 2.7.6a (PMID: 23104886) with –quantMode GeneCounts and –twopassMode Basic alignment settings. GENCODE (v22) was used for feature annotations. Genes with low expression were filtered out using edgeR’s function *filterByExpr()*. For survival modelling, raw counts were normalised using edgeR’s TMM (trimmed mean of M-values) method and log_2_ CPM (counts per million) transformed. ENSEMBL gene identifiers were annotated with Entrez gene identifiers using the R package org.Hs.eg.db (v3.10.0). Raw counts of two de novo metastatic samples (time = 0, event = 1) were excluded from survival analysis.

### Identification of differentially expressed and differentially variable genes

Genes whose mean expression in ILC samples were below the median of mean expression of Y-chromosome genes (log_2_ expression = 5.61) in both Metabric ILC and normal samples were assigned as unexpressed and filtered from the analysis. To identify differentially expressed genes between ILC (*n* = 148) and normal breast tissue samples (*n* = 144) in our discovery Metabric dataset, the R package limma (v 3.40.6) [30] was used. Significant genes were defined as those satisfying |log_2_ fold change| > 1 and FDR-adjusted *P* < 0.001.

To identify differentially variable genes between ILC samples and normal breast samples, we used the R package iDOS (v1.0.0) [31] and performed a test of variance between the two groups. Significantly variable genes were defined as genes with *σ*_ILC_ > *σ*_Normal_ and *σ*_ILC_ > 0.5 and FDR-adjusted *P* value < 1 × 10^−15^ (where *σ* represents standard deviation).

### Genes to pathways mapping

Over-representation analysis was performed on the differentially expressed and variable genes (1398) using MSigDB’s REACTOME dataset [32] to identify enriched pathways (Fisher’s exact test, R function: *phyper*). Pathways containing a minimum of three query genes and FDR-adjusted *P* < 0.1 were considered as statistically significant (*n* = 135 pathways). Genes (out of 1398) that did not map to the significantly enriched pathways were pooled together into a module of their own. To collapse overlapping pathways, we estimated a similarity measure using the overlap coefficient [33] between each pair of pathways and performed hierarchical clustering on the overlap coefficient matrix. Using the *cutree* function for *k* = 2 to 135, we calculated the average silhouette value [34] for each *k* with the R package cluster(v2.1.0) [35]. By maximising both silhouette value and the number of clusters (*k*), *k* was set to 30, resulting in 30 “cluster of pathways” (CP). Of note, some redundancy in these CPs existed to allow for functionally related pathways to be modelled together. These 30 CPs were subsequently used to create a database of modules for SIMMS [26] (R package: v1.3.1) which were tested for univariable prognostic value. Briefly, SIMMS shrinks a functionally related geneset (e.g. a CP) to those genes that are univariably associated with patient outcome (*P* < 0.05, Wald test). It then models these genes into a single per-patient risk score for each CP. For the creation of the multivariable Pathway-based Signature for ILC (PSILC) that aggregated all CPs, this database of 30 CPs was further refined to a non-redundant database of CPs by removing genes contributing to more than one CP. For each gene contributing to multiple CPs, we estimated its Spearman correlation coefficient (rho) against all other genes in a given CP and counted the number of instances of |rho| > 0.3. The per-CP correlated gene count was normalised by CP size, resulting in a measure of relevance (*R*) for a gene in a given CP. Each gene was assigned to the CP where it showed highest *R*. The resulting CPs were transformed into a SIMMS compatible non-redundant database of CPs used for multivariable modelling.

### Univariable (per CP) prognostic markers

The R package SIMMS (v1.3.1) was used to create univariable prognostic markers using the Metabric discovery cohort with feature selection threshold set to Wald test *P* value < 0.05 for the Cox proportional hazards model. Survival data was truncated at 10 years. mRNA abundance data was transformed to per gene *z*-scores (mean = 0, standard deviation = 1). Univariable models for CPs were applied to each of the six validation datasets to independently predict risk scores. These risk scores were dichotomised/trichotomised into risk groups using discovery set derived cut-offs; median for the two risk group classification and 33/66 percentiles for the three risk group classification. Risk groups from all six validation cohorts were combined and tested for association with patient outcome using the Cox proportional hazards model. For testing association with chemotherapy, risk groups from the SCAN-B cohort were assessed for prognostic and predictive association independently. For SCAN-B predictive evaluation (risk group × chemotherapy interaction), the model was adjusted for clinical covariates and tumour purity by modelling age as a dichotomous variable (>55 years: old, ≤55: young), T-stage as factors (0–2 cm tumour size: T1, 2–5 cm: T2, >5 cm: T3), nodal status as a dichotomous variable (0: negative, ≥1: positive) and tumour purity as a continuous variable. Significance of difference in survival curves was estimated using the Wald test (two risk group classification) and trend test (three risk group classification).

### Multivariable prognostic marker (PSILC)

Based on the non-redundant database of CPs (see “Methods: Genes to pathways mapping”), a multivariable model (PSILC) was created using the Random Forest algorithm with R packages SIMMS (v1.3.1) and randomForestSRC (v2.11.0). The Metabric discovery cohort (*n* = 148) was split into 66% training and 33% internal cross-validation. Survival time was truncated at 10 years. In addition we performed a parameter sweep for a number of hyperparameters including *ntrees*: 501 to 1001 (step size of 100), *nodesize*: 10 to 15 (step size of 1), *mtry*: sqrt(30) to 30/3 i.e. 5 to 10 (step size of 1). We picked the top model with lowest out-of-bag (OOB) error and highest *β* estimated from the Cox proportional hazards model. The resulting model (PSILC) was based on ntrees = 601, nodesize = 10 and mtry = 10. PSILC was applied to each of the six validation datasets independently to predict risk scores. These risk scores were dichotomised/trichotomised into risk groups using discovery cohort derived cut-offs; median for the two risk group classification and 33/66 percentiles for the three risk group classification. Risk groups from all six validation cohorts were tested for association with patient outcome using the Cox proportional hazards model, both independently in each dataset as well as in a combined validation cohort. Proportional hazards assumption was evaluated using the Schoenfeld test (R function *survival::cox.zph()*). We failed to reject the null hypothesis that hazards are proportional where *P* > 0.05 for the assessed coefficient. Significance of difference in survival curves was estimated using the Wald test (two risk group classification) and trend test (three risk group classification). For the Guedj dataset (two risk group classification), we used the log-rank test, owing to no events in its predicted low-risk group. Adjustment for clinical covariates and tumour purity was performed by modelling age as a dichotomous variable (>55 years: old, ≤55: young), T-stage as factors (0–2 cm tumour size: T1, 2–5 cm: T2, >5 cm: T3), nodal status as a dichotomous variable (0: negative, ≥1: positive) and tumour purity as a continuous variable.

### Purity estimation

Purity for each dataset was estimated using the R package estimate (v1.0.13).

### Signature comparison

Mammaprint, Oncotype DX and PAM50 ROR risk scores were estimated using the implementation in the genefu (v2.16.0) R package [36]. The ILC-specific LobSig [9] signature was applied using the published code from GitHub repository https://samirlal2.github.io/LobSig/. For the LobSig two risk group classification, the Metabric-derived median score was used to dichotomise risk scores into high- and low-risk groups in the six validation datasets. For the LobSig three risk group classification, the Metabric-derived 33% and 66% percentiles of risk scores were used to split validation datasets into three risk groups. Concordance between the risk groups derived from different biomarkers was evaluated using the Cohen’s kappa statistics with R package fmsb (v0.7.0). To compare predictive power of different biomarkers on the combined validation cohort, −log_10_ *P* values (Wald test or trend test) were estimated from the Cox proportional hazards model fitted to risk groups predicted by each biomarker. To account for unbalanced size of validation cohorts, we also compared Stouffer’s weighted *P* values of biomarkers using the R package metap (v1.3).

### CRISPR perturbation screens analysis

For the 17 ILC or ILC-like cell lines, matched RNA-Seq data and CRISPR-Cas9 gene essentiality profiles were downloaded from the DepMap portal (https://depmap.org/portal/depmap/, version: 21Q4). Cell line mutation data and amplification status of *ERBB2* was curated from the DepMap portal. Copy-number deletions for *PTEN* were curated from cBioPortal (CCLE Broad, 2019). Statistical analysis assessing CRISPR gene effect scores between the PSILC high vs. low-risk scores was performed using a two sample one-sided Welch’s *t*-test where a minimum of 3 observations per group were available. Statistical testing was restricted to genes satisfying the following gene effect (GE) scores criteria:

$$\left\{\begin{array}{c}{{{{{{\rm{mean}}}}}}}\left({{{{{{\rm{GE}}}}}}}\,{{{{{{\rm{score}}}}}}}\,{{{{{{\rm{in}}}}}}}\,{{{{{{\rm{PSILC}}}}}}}\,{{{{{{\rm{high}}}}}}}\,{{{{{{\rm{group}}}}}}}\right) < -0.5\\ {{{{{{\rm{mean}}}}}}}\left({{{{{{\rm{GE}}}}}}}\,{{{{{{\rm{score}}}}}}}\,{{{{{{\rm{in}}}}}}}\,{{{{{{\rm{PSILC}}}}}}}\,{{{{{{\rm{low}}}}}}}\,{{{{{{\rm{group}}}}}}}\right) > -0.75\\ {{{{{{\rm{mean}}}}}}}\left({{{{{{\rm{GE}}}}}}}\,{{{{{{\rm{score}}}}}}}\,{{{{{{\rm{in}}}}}}}\,{{{{{{\rm{PSILC}}}}}}}\,{{{{{{\rm{high}}}}}}}\,{{{{{{\rm{group}}}}}}}\right) < {{{{{{\rm{mean}}}}}}}\left({{{{{{\rm{GE}}}}}}}\,{{{{{{\rm{score}}}}}}}\,{{{{{{\rm{in}}}}}}}\,{{{{{{\rm{PSILC}}}}}}}\,{{{{{{\rm{low}}}}}}}\,{{{{{{\rm{group}}}}}}}\right)\\ \,\end{array}\right.$$

Genes with a median gene effect score of <−1 considering all breast cancer cell lines were regarded as essential genes and subsequently annotated/excluded from the analysis, post-hoc.

### Data processing, statistical analyses and visualisations

All data processing, statistical analyses, and plotting were performed in the R statistical environment (v3.6.0).

## Results

### Dysregulated genes in ILCs comprise prognostic candidates and encompass hallmarks of cancer

**Fig. 1:**
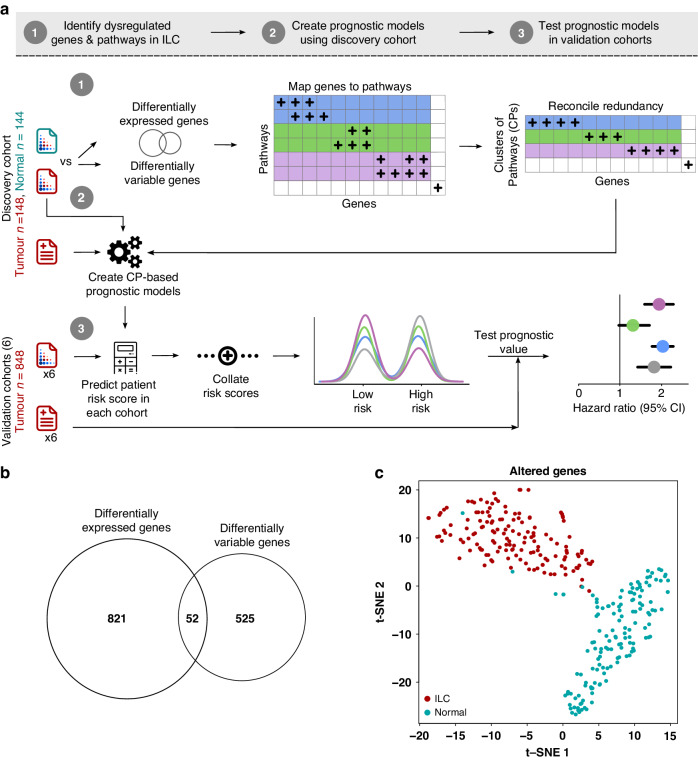
Identification of dysregulated and prognostic genes/pathways in ILCs. **a** Schematic of the methodology, divided into 3 steps. Step 1 constitutes feature selection using the Metabric discovery cohort with mRNA abundance profiles of ILC (*n* = 148) and normal breast tissue samples (*n* = 144). ILC and normal mRNA abundance profiles were compared by performing differential gene expression and differential variance analysis. The resulting genes were subject to pathway over-representation analysis to identify dysregulated pathways in ILCs. To remove pathway redundancy, pathways with shared genes due to nested hierarchy were collapsed into pathway modules called a “cluster of pathways” (CP). In step 2, a survival model for each CP was created using Metabric mRNA abundance profiles and clinical outcome data. In step 3, each CP-based survival model was applied to the mRNA abundance profiles of the six validation cohorts separately to predict patient risk score. These predicted risk scores were dichotomised into risk groups using the discovery cohort’s median. The resulting risk groups from six validation cohorts were combined and subsequently correlated with patient outcome. **b** Venn diagram showing the overlap of differentially expressed and differentially variable genes between ILC (*n* = 148) and normal breast tissue samples (*n* = 144), identified using the Metabric dataset. **c** t-SNE clustering of tumour and normal samples using 1398 ILC-dysregulated genes.

To identify prognostic biomarkers of lobular breast cancer, we designed a data analysis workflow that exploited transcriptomic profiles of the disease (Fig. 1a). We curated seven independent retrospective breast cancer cohorts comprising 996 ILCs with mRNA abundance and outcome data (Supplementary Table 1). Of these, the Metabric cohort (*n* = 148) was designated as the discovery cohort, given its availability of normal breast samples (*n* = 144) and long-term outcome data with median follow-up time of 12.26 years. Using the Metabric cohort, dysregulated genes in ILCs were identified using two complementary approaches. First, differentially expressed genes in ILC patients were identified by comparing the difference in mean mRNA abundance between tumour and normal samples (873 genes, |log_2_FC| > 1, FDR-adjusted *P* < 0.001; Supplementary Table 2). Second, genes that showed increased mRNA abundance heterogeneity in ILC patients were identified by comparing the difference in standard deviation between ILC and normal samples (577 genes, *σ*_ILC_ > *σ*_Normal_, *σ*_ILC_ > 0.5, FDR-adjusted *P* < 10^−15^; Supplementary Table 3). Next, we combined these two lists to create a resource of 1398 candidate ILC-dysregulated genes (Fig. 1b, c). Of these, 52 genes were predictive of overall survival in the discovery cohort, with 10 and 42 genes associated with poor and good outcome, respectively (FDR-adjusted *P* < 0.05; Supplementary Table 4). To evaluate ILC-specificity of these 52 genes, we tested the prognostic power of ILC-dysregulated genes in the ER+/HER2− IBC-NST samples of the Metabric cohort (*n* = 1046). Although there were higher numbers of prognostic genes in ER+/HER2− IBC-NST (187 genes, FDR-adjusted *P* < 0.05), likely due to increased statistical power, only 12 were in common with the 52 prognostic genes identified in ILC samples (Supplementary Fig. 1). In order to functionally interpret the ILC-dysregulated gene list we performed a pathway over-representation analysis, resulting in 135 significantly enriched pathways (FDR-adjusted *P* < 0.1; Supplementary Table 5). These pathways encompassed a variety of canonical and non-canonical cancer pathways as well as key hallmarks of cancer such as cell cycle, metabolism, immune system and signal transduction; suggesting a wide-spread transcriptomic dysregulation in ILC patients.

### Dysregulated pathways in ILC predict patient outcome and reveal association with chemotherapy response

**Fig. 2:**
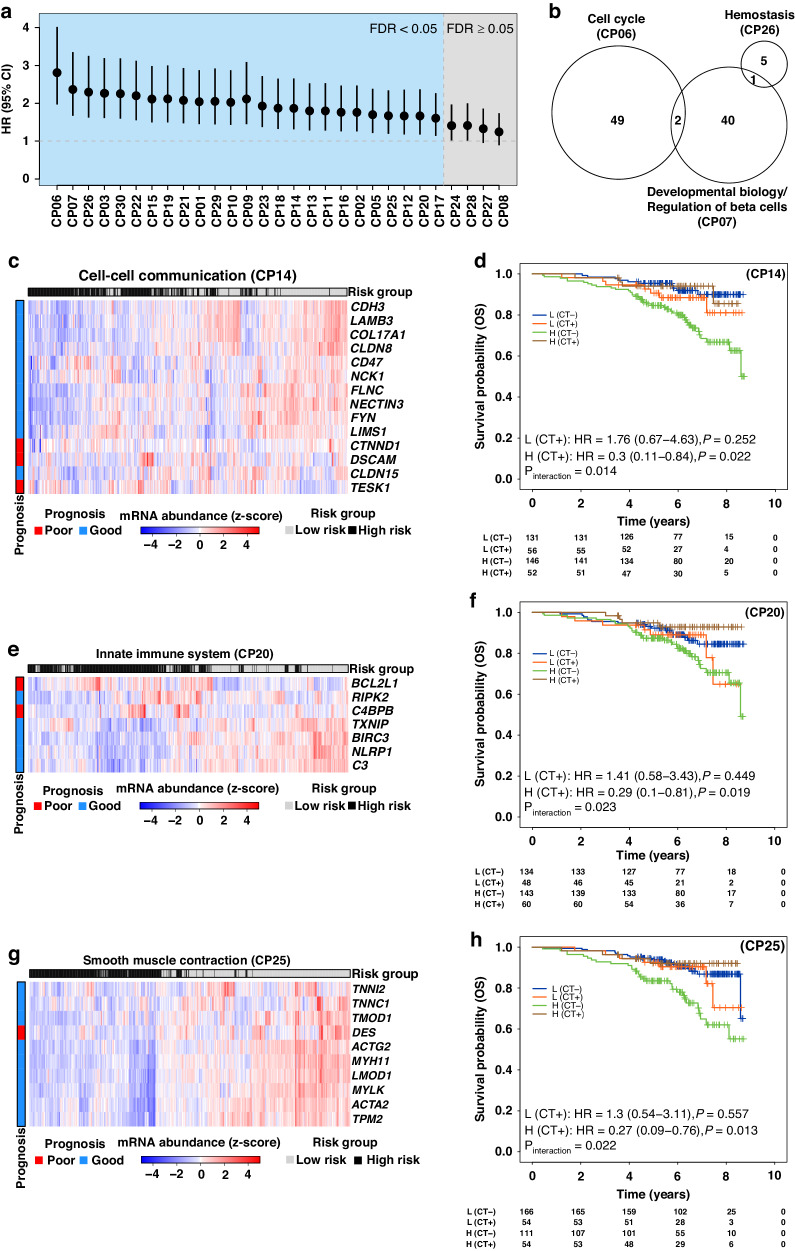
Univariable prognostic models. **a** Forest plot showing prognostic assessment of CPs in the combined validation cohort (HR, 95% CI are shown). Predicted risk scores for CPs in the combined validation cohort were dichotomised into low- and high-risk groups using the discovery cohort’s median risk score for the corresponding CP. CPs with significant prognostic association are left of the vertical reference line in blue section (FDR-adjusted *P* < 0.05). **b** Venn diagram of prognostic genes from the top 3 CPs. **c** Heatmap showing mRNA abundance profiles of prognostic genes from CP14 in combined validation cohorts. Row covariate indicates whether a gene was associated with poor or good outcome in univariable analysis in the discovery cohort. Column covariate indicate predicted risk group in the combined validation cohort. Hierarchical clustering was performed on both rows and columns using “euclidean” as the distance measure and “ward” as the agglomeration method. **d** Kaplan–Meier survival curves (overall survival (OS)) indicating prognostic assessment of CP14 stratified by chemotherapy response using a contemporary clinical cohort (SCAN-B). **e**–**h** Same as (**c**, **d**), showing mRNA abundance profiles of prognostic genes from CP20 and CP25 and their prognostic assessment stratified by chemotherapy.

To rationalise clinical heterogeneity in ILC outcome at a molecular level, pathways enriched for ILC-dysregulated genes were tested for prognostic potential. Prior to prognostic assessment, highly redundant enriched pathways were collapsed into cluster of pathways (CP) (Supplementary Fig. 2a, b, Supplementary Table 6; “Methods: Genes to pathways mapping”). While some overlap between CPs remained in order to allow complete coverage of functionally related genes in a CP, the resulting dataset represented a reduced set of 29 functionally distinct CPs. Genes that did not map to any pathway during the enrichment analysis were designated to their own cluster (CP30). To evaluate the prognostic potential of each CP, we used a previously published algorithm (SIMMS) [26] for quantifying pathway dysregulation. Using the discovery cohort (Metabric cohort [37]), a univariable Cox proportional hazards model for each CP was created (time to event: overall survival) and subsequently tested in a combined validation cohort from six independent studies (Supplementary Table 1). The prognostic evaluation in the combined validation cohort revealed 25 CPs significantly associated with patient outcome (FDR-adjusted *P* < 0.05, Wald test; Fig. 2a and Supplementary Table 7). The top three prognostic CPs included Cell cycle (HR = 2.81, 95% CI = 1.97–4.02, FDR-adjusted *P* = 4.09 × 10^−7^), Developmental biology/regulation of beta cells (HR = 2.34, 95% CI = 1.67–3.36, FDR-adjusted *P* = 1.95 × 10^−5^) and Haemostasis (HR = 2.3, 95% CI = 1.62–3.26, FDR-adjusted *P* = 2.33 × 10^−5^). These top three prognostic CPs showed minimal overlap of prognostic genes, confirming the presence of independent prognostic metagenes between them (Fig. 2b).

Some ILCs are less responsive to chemotherapy and molecular mechanisms associated with chemoresistance remain unclear [38]. Hence, we asked whether prognostic CPs could reveal molecular pathways associated with chemotherapy response. Using a contemporary clinical cohort (SCAN-B) [27], which represents the latest clinical chemotherapeutic modalities, we tested prognostic CPs in chemotherapy naïve and chemotherapy treated subgroups. Of the significantly prognostic CPs, 17 out of 25 CPs remained prognostic in the chemotherapy naïve subgroup (FDR-adjusted *P* < 0.05, Supplementary Table 8), while none of the CPs were prognostic in the chemotherapy treated subgroup (Supplementary Table 9). This suggests that CP-predicted high-risk patients in the chemotherapy naïve group could benefit from chemotherapy. To formally test this observation, we stratified patient risk groups by chemotherapy status and evaluated the interaction term (risk group × treatment) of the model, limiting to the 17 CPs that were prognostic in the chemotherapy naïve subgroup. Of these, four CPs (CP14: Cell-cell communication, CP20: Innate immune system, CP25: Smooth muscle contraction and CP10: Extracellular matrix organisation) were predictive of potential benefit from chemotherapy in the high-risk group (Interaction *P* < 0.05, Fig. 2c–h and Supplementary Table 10). Three of these (CP14, CP20 and CP25) remained independent predictors when adjusted for clinical covariates in a multivariable model (Interaction *P* < 0.05, covariates: age, T-stage, nodal status, tumour purity; “Methods: Univariable (per CP) prognostic markers”).

### Aggregating multiple pathways reveals a robust biomarker of patient outcome

**Fig. 3:**
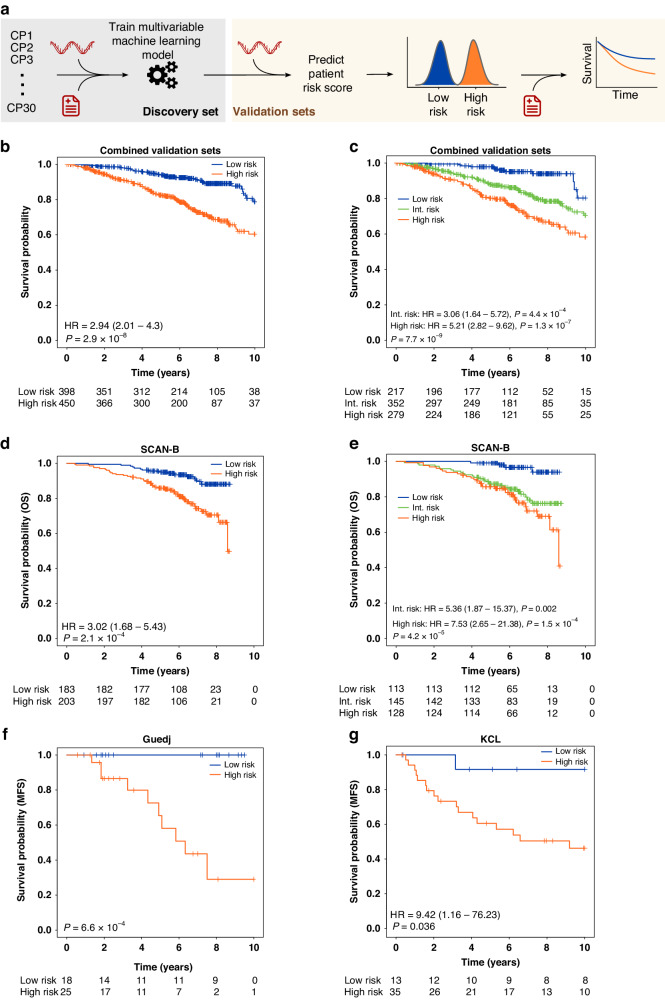
Multivariable prognostic model (PSILC). **a** Schematic of the methodology for discovery and validation of the multivariable survival model PSILC. Allocation of discovery and validation cohorts was maintained as defined in Fig. 1 and Supplementary Table 1. **b**, **c** Kaplan–Meier survival curves indicating prognostic assessment of PSILC in the combined validation cohort using two and three risk group classifications. **d**, **e** Kaplan–Meier survival curves indicating prognostic assessment of PSILC in the contemporary SCAN-B dataset assessing overall survival using the two and three risk group classifications. **f** Kaplan–Meier survival curves indicating prognostic assessment of PSILC in the Guedj dataset assessing metastasis-free survival (MFS) using the two risk group classification. Hazard ratios are not reported due to lack of events in the reference group (low-risk). **g** Kaplan–Meier survival curves indicating prognostic assessment of PSILC in an independent lobular cancer dataset (KCL), assessing metastasis-free survival using the two risk group classification. Survival analysis was adjusted for age, T-stage, and nodal status.

Building on the prognostic capabilities of CPs, we created a multivariable signature by aggregating [26] multiple CPs (30 variables) into a machine learning model using the Random Forest algorithm (Fig. 3a). This multivariable Pathway-based Signature for ILC (PSILC) was trained and cross-validated using the Metabric discovery cohort (time to event: overall survival), and subsequently applied to six independent validation cohorts (Supplementary Fig. 3; “Methods: Multivariable prognostic marker”). Using the discovery cohort derived median risk score cut-off, PSILC was able to successfully classify patients into appropriate risk groups in the combined validation cohort (HR = 2.94, 95% CI = 2.01–4.3, *P* = 2.9 × 10^−8^; Fig. 3b and Supplementary Table 11). The 10-year survival for the predicted high-risk group was 60.41% compared to 78.8% in the low-risk group. The predicted risk groups were distinctively correlated with pathway scores and confirmed the presence of at least two predominant clusters of patients, independent of dataset and clinical covariates (Supplementary Fig. 4). To allow for the presence of additional distinct sub-groups, in the absence of clinically relevant risk score cut-offs to define patient groups, we further assessed prognostic relevance of PSILC predicted risk scores by creating three risk groups based on discovery cohort’s tertiles. This classification demonstrated an increased separation between the low- and high-risk groups (HR = 5.21, 95% CI = 2.82–9.62, *P* = 1.3 × 10^−7^; Fig. 3c and Supplementary Table 11) while the outcome of the intermediate-risk group was also significantly poor compared to the low-risk group (HR = 3.06, 95% CI = 1.64–5.72, *P* = 4.4 × 10^−4^; Fig. 3c). The 10-year survival in the predicted high- and low-risk groups was 58.3% and 80.28%, respectively. PSILC remained an independent prognostic biomarker when adjusted for age, T-stage, nodal status and tumour purity (two group classification: *P* = 4.8 × 10^−6^, three group classification: *P*_Trend_ = 3.1 × 10^−6^; Supplementary Table 12). Next, we separately evaluated PSILC in a contemporary clinical cohort (SCAN-B, *n* = 386) where the time to event data (overall survival) was influenced by the most up-to-date treatment strategies. In the SCAN-B cohort, PSILC showed robust prognostic ability in classifying patients into appropriate outcome groups (two group classification: HR = 3.02, 95% CI = 1.68–5.43, *P* = 2.1 × 10^−4^, three group classification: HR_High vs. Low_ = 7.53, 95% CI = 2.65–21.38, *P* = 1.5 × 10^−4^; Fig. 3d, e). We further assessed whether the predicted high-risk group was simply a proxy for the aggressive subtypes of breast cancer triple negative breast cancer (TNBC) or HER2-positive cancer. In the SCAN-B cohort, 18 ILCs (4.66%) were either TNBC (*n* = 6) or HER2-positive (*n* = 12) which is not unexpected since classic ILCs are predominantly ER+/Luminal-A and HER2-negative breast cancers [2]. Of these 18 TNBC/HER2-positive patients, five were classified as low-risk and 13 as high-risk by PSILC. When PSILC multivariable model was adjusted for TNBC/HER2 status, it remained an independent predictor of overall survival (two group classification: HR = 2.92, 95% CI = 1.62–5.25, *P* = 3.6 × 10^−4^). PSILC also demonstrated potential in predicting benefit from chemotherapy in the high-risk group (HR = 0.52, 95% CI = 0.24–1.13, *P* = 0.099) while this was lost in the low-risk group (Supplementary Fig. 5a). Having previously demonstrated ILC-specificity of prognostic genes in a univariable context (Supplementary Fig. 1), here we asked whether the multivariable PSILC was specific to ILCs by testing it in 1795 patients from the SCAN-B cohort that were IBC-NST and ER+/HER2-negative. PSILC-predicted risk groups in this subset showed moderate separation (HR = 1.93, 95% CI = 1.46–2.57, *P* = 5.4 × 10^−6^; Supplementary Fig. 5b) and markedly inferior performance compared to the ILC subset of SCAN-B (Concordance index: IBC-NST/ER+/HER2− = 0.58 (95% CI = 0.54–0.62), ILC = 0.65 (95% CI = 0.59–0.72)), highlighting ILC-specificity of PSILC.

While treatment strategies for ILCs are similar to IBC-NST, ILCs have distinct metastatic patterns compared to IBC-NST [1, 39]. Hence, we tested PSILC in Guedj et al.’s cohort [40] (*n* = 43) where time to event data was based on metastasis-free survival (MFS). In this cohort alone, PSILC accurately predicted the likelihood of metastasis (two group classification: *P* = 6.6 × 10^−4^, Fig. 3f). To further validate PSILC’s ability in predicting MFS, we performed RNA sequencing on a series of 48 archival lobular breast cancers (treated at the Guys Hospital, London; KCL cohort) with a median follow-up of 17.75 years. This series was predominantly composed of pleomorphic lobular cancers (PLCs) that are considered a highly aggressive form of ILCs [41, 42]. All the patients in the KCL cohort were grade 3 lobular cancers and 37.5% had a metastatic event within 10 years of diagnosis (Table 1). When PSILC was tested in this cohort, the resulting classification showed strong association with MFS independent of age, T-stage and nodal status (HR = 9.42, 95% CI = 1.16–76.23, *P* = 0.036, Fig. 3g and Supplementary Table 13). Given the metastatic tendency of PLCs, we examined the breakdown of PSILC risk groups by known aggressive subtypes of breast cancer (TNBC and HER2-positive) to understand the added value of PSILC in predicting MFS. Of the 12 TNBC or HER2-positive patients in the KCL cohort, 11 were classified as high-risk, confirming accurate risk assignment of these patients by PSILC (HR = 7.52, 95% CI = 0.99–57.35, *P* = 0.052; when adjusted for TNBC/HER2 status). Additionally, there were 24 patients in the high-risk group that had poor outcome but were neither TNBC nor HER2-positive, further emphasising PSILC’s ability to capture molecular changes that are conserved across aggressive ILCs but independent of TNBC/HER2 status.

**Table 1:** Clinical and pathological characteristics of KCL cohort.

	Number (*n*)
ILC tumour samples	48
Pleomorphic	45
Classic	3
Average patient age (years)	59.5
Disease relapse status	
No Relapse	25
Relapse	23
<3 years of primary diagnosis	9
3–6 years after primary diagnosis	6
>6 years after primary diagnosis	7
Onset unknown	1
Tumour size	
≤20 mm	12
>20 mm, ≤50 mm	27
>50 mm	9
Number of involved lymph nodes	
0	19
1–3	15
4–9	10
≥10	4
Sites of metastases	
Bone	13
Liver and peritoneum	2
Abdomen	2
Pleura	2
Skin	2
Lung	1
Brain	1
Soft tissue	1
Brachial Plexopathy	1
Unknown	4
Tumour grade	
III	48
Oestrogen (ER) status	
ER+ve	37
ER-ve	7
Unknown	4
Progesterone (PR) status	
PR+ve	30
PR-ve	11
Unknown	7
HER2 status	
HER2+ve	7
HER2-ve	37
Unknown	4

**Fig. 4:**
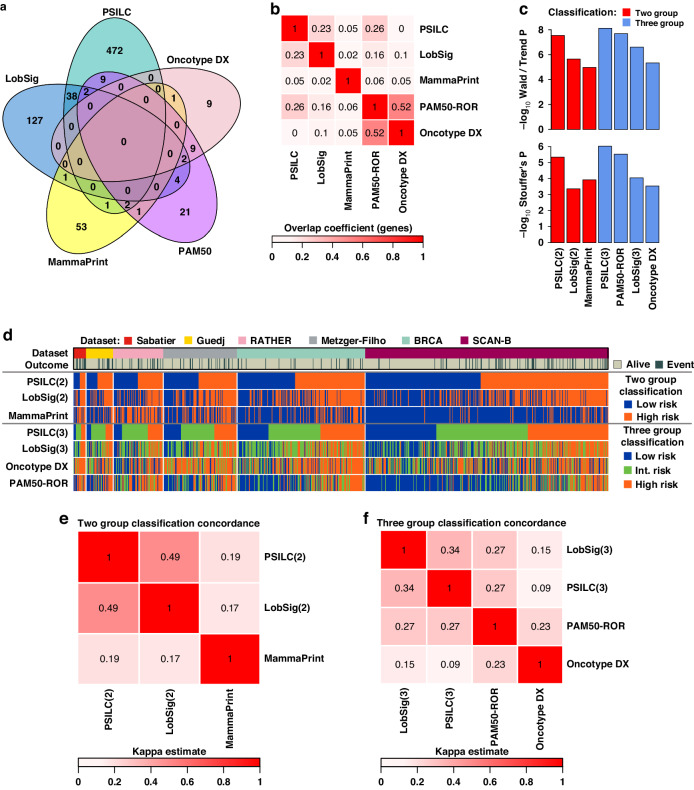
Signature comparison. **a** Venn diagram showing overlap between genes from PSILC and other breast cancer risk signatures: LobSig, PAM50, Oncotype DX and MammaPrint. **b** Heatmap of overlap coefficient between genes from PSILC and other breast cancer risk signatures. A higher overlap coefficient indicates a greater overlap. **c** Comparative evaluation of PSILC’s two risk group classification with LobSig(2) and MammaPrint, and the PSILC three risk group classification with LobSig(3), PAM50 and Oncotype DX in matched validation cohorts. −log_10_*P*-values of combined validation cohort (top panel) and cohort-wise −log_10_*P*-values combined using the Stouffer’s method (bottom panel, to account for unbalanced size of validation cohorts) were compared. Wald test was used for the two risk group classification and trend test was used for the three risk group classification. **d** Concordance plot showing predicted risk groups by PSILC and other breast cancer risk signatures. Column covariates show validation datasets and survival events. Plot is ordered by PSILC risk scores in each validation cohort separately. Heatmaps showing concordance (expressed as *Kappa* estimate) between the risk groups predicted by the PSILC two risk group (**e**) and three risk group (**f**) classifications, and other breast cancer risk signatures. Higher Kappa values indicate greater concordance.

To benchmark prognostic potential of PSILC, a panel of four independent prognostic biomarkers were tested in the same six validation cohorts that were used for testing PSILC. These biomarkers included three clinically approved breast cancer risk predictors (MammaPrint [43], Oncotype DX [15] and PAM50 risk of recurrence score (PAM50-ROR) [22]), and one lobular breast cancer biomarker (LobSig [9]). A comparison of genes involved in these signatures revealed only a partial overlap, with most of the genes exclusive to one signature (PSILC overlap coefficient with other signatures: 0–0.26, Fig. 4a, b). This is consistent with previous studies that have shown the presence of large numbers of valid signatures in breast cancer [44], as well as pancreatic [45] and lung cancer [46]. Next, using two independent measures to quantify prognostic significance (Wald or Trend *P*, Stouffer’s weighted *P*; “Methods: Signature comparison”), we compared the prognostic ability of PSILC’s two group and three group classifiers with the corresponding two group (LobSig and MammaPrint) and three group classifiers (LobSig, PAM50-ROR, Oncotype DX) of other biomarkers. Although all biomarkers were able to classify patients into significantly different outcome groups (*P* < 0.001), PSILC showed superior performance in both the two group and the three group classification systems, with PAM50-ROR’s performance remaining competitive to PSILC in the three group classification (Fig. 4c). Further inspection of the predicted risk groups revealed substantial misclassification heterogeneity between the biomarkers in both the two group and the three group classification systems (Fig. 4d). The ILC-specific predictors (PSILC and LobSig) showed the highest, albeit modest, concordance in predicted risk groups (two group classification: Cohen’s kappa = 0.49, three group classification: Cohen’s kappa = 0.34; Fig. 4e, f).

### Multivariable pathway model (PSILC) reveals context-specific genetic dependencies in aggressive ILCs

**Fig. 5:**
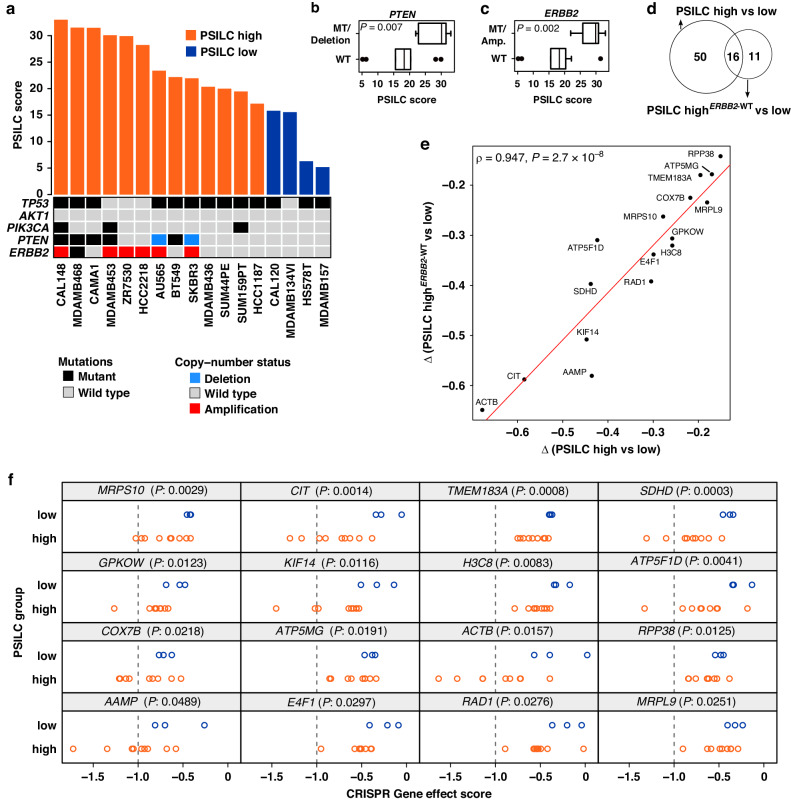
Identification of candidate vulnerabilities in PSILC high group. **a** Bar plot of PSILC predicted scores in ILC/ILC-like breast cancer cell lines. PSILC high and low groups were inferred from predicted scores using the threshold derived from the risk scores of the Metabric discovery cohort. Cell line mutation profiles for *TP53, AKT1, PIK3CA, PTEN* and *ERBB2* and copy-number profiles for *PTEN* and *ERBB2* are shown below the bar plot. PSILC scores in cell lines grouped by *PTEN* status (**b**, mutant (MT)/Deleted vs. wild type) and *ERBB2* status (**c**, mutant (MT)/Amplified vs. wild type). Significance between PSILC scores (“P”: *P* value) was estimated using a two sample Welch’s *t*-test. **d** Overlap between significant synthetic lethal genes identified in two separate analyses (with- and without *ERBB2* altered samples). **e** Scatter plot showing correlation between the mean difference (∆) of shared synthetic lethal genes (see **d**) identified in two separate analyses (with- and without *ERBB2* altered samples). Red line shows linear regression fit. **f** CRISPR gene effect profiles of high-confidence synthetic lethal genes. Dashed line indicate threshold applied to gene effect scores to exclude essential genes. *P* values were estimated using the two sample one-sided Welch’s *t*-test.

Despite differences in the underlying biology and response to endocrine therapy between ILC and IBC-NST [16, 47, 48], there are no clinically approved targeted therapies for ILCs that exploit these differences. One approach towards developing targeted therapies is to identify genes that are essential for cancer cell fitness and survival using CRISPR/Cas9 genetic perturbation screens [49]. We therefore used publicly available transcriptomic profiles from 17 ILC or ILC-like [3] breast cancer cell lines using the CCLE [50] dataset, classified each cell line into PSILC high and low groups based on the threshold derived from the previously used PSILC discovery dataset (Metabric) and then used genome-wide CRISPR/Cas9 screen data from these same cell lines (derived from DepMap.org) to identify genes that when CRISPR-Cas9 targeted, selectively reduced viability in the PSILC high group (Fig. 5a). Most (13 out of 17) cell lines were classified as PSILC high, which was not surprising since cell lines are, in general, derived from aggressive disease. We further assessed the potentially confounding impact of key breast cancer driver genes on PSILC classifications. While *TP53* mutations were present in 13 out of 17 cell lines, *AKT1* and *PIK3CA* mutations were rare. However, cell lines with high PSILC score were frequently *PTEN* inactivated and/or harboured an *ERBB2* amplification (*P*_PTEN_ = 0.007, *P*_ERBB2_ = 0.002, Fig. 5b, c; Welch’s *t*-test), both of which are known features of aggressive breast cancers [51, 52] and *ERBB2-*targeted therapies are already in clinical use [53]. To derive candidate synthetic lethal genes in PSILC high scoring cell lines while accounting for the known vulnerabilities of *ERBB2* amplified cancers, we compared DepMap’s aggregated CRISPR-Cas9 gene effect profiles [54] between (1) PSILC high vs. low group, and (2) PSILC high^*ERBB2*-WT^ vs. low group (Supplementary Tables 14 and 15, “Methods: CRISPR perturbation screens analysis”). Briefly, lower gene effect score (<0) indicates the likelihood of a gene’s essentiality with zero being not essential and -1 being the median of essential genes [54]. The resulting list of candidate synthetic lethal genes in PSILC high scoring lines from the two analyses (with and without *ERBB2* altered cell lines) revealed 66 and 27 genes, respectively, that when targeted by CRISPR-Cas9, selectively decreased viability in the PSILC high group (*P* < 0.05, one-sided Welch’s *t*-test). Sixteen genes were found in both analyses and were therefore considered independent of *ERBB2* status (a statistically significant overlap: *P* = 3.27 × 10^−33^, Fisher’s exact test; Fig. 5d). As expected, these 16 genes demonstrated strong correlation (Spearman’s *ρ* = 0.947, *P* = 2.7 × 10^−8^) in the difference of their mean gene effect scores (∆) for both analyses (PSILC high vs. low and PSILC high^*ERBB2*-WT^ vs. low group) (Fig. 5e, f). Pathway over-representation analysis of these 16 candidate synthetic lethal genes revealed significant enrichment in metabolic pathways (*SDHD, ATP5F1D, ATP5MG* and *COX7B*), Rho GTPases (*CIT, H3C8, KIF14* and *ACTB*) and Haemostasis (*H3C8, ACTB* and *AAMP*) (Supplementary Table 16, FDR-adjusted *P* < 0.05; Fisher’s exact test). With the exception of *H3C8*, 15 out of 16 genes were also expressed (mRNA abundance: mean log_2_ (transcripts per million + 1) > 3 in PSILC high group) at moderate to high-levels and therefore further supports their targeting potential. Together, these findings highlight therapeutic potential of these candidate synthetic lethal genes which would require focussed interrogation by drug screening assays in future studies.

## Discussion

Invasive lobular breast cancer is recognised as a distinctive histological subtype of breast cancer [1, 39]. However, systematic discovery and independent validation of biomarkers specific for this disease remains limited [9]. Here, we assessed prognostic and predictive value of dysregulated pathways in ILC through meta-analysis of seven gene expression studies. While several pathways were prognostic, Cell-cell communication, Innate immune system and Smooth muscle contraction pathways were independent predictors of response to chemotherapy in the predicted high-risk group. Patients with a higher risk score, estimated using these pathways, showed significantly better overall survival when prescribed chemotherapy compared to chemotherapy naïve patients, thereby highlighting the potential benefit of treatment escalation for predicted high-risk patients.

In a multivariable setting where a machine learning approach was employed to create a multi-pathway predictor “PSILC”, we showed the strong prognostic potential of pathways altered in ILCs for both overall survival and metastasis-free survival. Although it is recognised that ILCs have distinctive metastatic patterns compared to IBC-NST [2, 6], it remains unclear which tumours would eventually metastasise. Focussing on this unmet need, we RNA sequenced an independent clinical cohort that largely comprised of highly aggressive grade 3 pleomorphic lobular cancers [41, 42], where PSILC was able to accurately estimate the risk of 10-year metastasis with baseline mRNA profiles. This result, although with limitations in statistical power, highlights the utility of PSILC as a tool to identify patients where current treatment options are unlikely to succeed, especially in the long-term, which remains a clinical challenge in the management of ILCs compared to IBC-NST [6].

A number of studies have identified gene expression-based molecular subtypes of ILCs. However, independent validation of the prognostic capacity of these subtypes remains limited [5, 9, 23]. LobSig [9], a supervised signature of ILCs was validated in one independent study (RATHER consortium [23]). The RATHER consortium and TCGA [5] took a gene expression-based clustering approach to identify two and three subtypes, respectively, with a substantial overlap in the underlying biology of the two classification schemes. While RATHER subtypes were reproducible in TCGA [5] and Metabric [37] studies, these subtypes were not associated with clinical outcome. The TCGA three risk group classifier-identified subtypes [5] (Reactive-like, Immune-related and Proliferative), were also reproducible in TCGA and Metabric studies and showed significantly poor outcome for the Proliferative subtype compared to the Reactive-like in the Metabric study. Here, we systematically evaluate a panel of breast cancer biomarkers where classifiers were available, including three clinically approved tests across six ILC validation studies. While PSILC showed highest prognostic ability, PAM50-ROR [22] was also a strong predictor of outcome, which is consistent with successful testing of PAM50-ROR in a population based ILC cohort [55]. Importantly, none of the three clinical tests evaluated in this study (PAM50-ROR, Oncotype DX and MammaPrint) were developed to capture ILC-specific biology or to predict late recurrences, which is a crucial end-point for ILCs [6]. Specifically, Oncotype DX and MammaPrint were developed for ER+ breast cancers. Therefore, PSILC addresses a critical need to develop new tests that delineate the biology of ILCs in order to successfully convert prognostic groups into drug discovery and treatment strategies aimed at this histological subtype of breast cancer. As seen here, across the above-mentioned biomarkers, genes showed limited inter-biomarker overlap, highlighting a potential impact on the selection of candidate targets suitable for designing treatment strategies for prognostic groups.

A key limitation to identifying an accurate and robust biomarker is a lack of adequately powered studies. For instance, ILCs constitute up to 15% of breast cancers, and therefore even the largest available breast cancer cohorts with matched genomic data [5, 27, 37] are underpowered for the detection and validation of new biomarkers. This limitation is further exacerbated by the fact that these cancers frequently have late recurrences [6] which necessitate long-term follow-up studies. Here, although we make use of all available studies with survival data (Supplementary Table 1), these limitations are likely to impact our results. To minimise this impact, we have performed the discovery of signatures using the Metabric study [37] which has the longest median follow-up (>10 years for ILC samples) of all available clinico-genomic studies of breast cancer, and further perform validation in a new in-house cohort (KCL, *n* = 48) with a median follow-up of 17.75 years. Furthermore, our results are likely sensitive to differences in treatment decisions within the eight cohorts studied here. Therefore, prospective studies with appropriate statistical power and long-term follow-up survival data would be necessary, not only to test the clinical suitability of the prognostic signatures derived here, but also for the development of new signatures to match the specific genetic background of ILC sub-populations, such as ~13% of ILCs that harbour *PTEN* loss [5].

While accurate prognostication remains a key clinical question, it is also crucial to match prognostic subgroups with the right drug to deliver precision medicine. Here we propose a novel workflow illustrating how this can be achieved by applying the multivariable prognostic biomarker “PSILC” to breast cancer ILC/ILC-like cell lines. For these cell lines, we delineate 16 context-specific (selective in high PSILC score) candidate synthetic lethal interactions. Some of these genes are annotated as key essential genes in pan-cancer cell line studies [54], which needs to be acknowledged, as essential genes are often not considered as valid synthetic lethal targets. However, we have considered essentiality in a breast cancer specific manner and more importantly contextualised it alongside selectivity [56], as successfully demonstrated in the case of ATR inhibitors in previous studies [57, 58]. Despite the success of cell lines and organoid models in cancer research, reliable models of lobular cancers of the breast remain limited [3] with most models designated as ILC-like. The selection of these models is often guided by the inactivation of E-cadherin and/or alpha-catenin, and low mRNA abundance of E-cadherin. Although this approach offers a panel of candidate cell lines potentially representative of patient biology, accurate in vitro and pre-clinical models recapitulating primary ILCs are critically needed [3]. Such models would be necessary to identify a precise biomarker-defined population suitable for prospective pre-clinical and early clinical work.

### Supplementary information

Supplementary Materials

Supplementary Table 01

Supplementary Table 02

Supplementary Table 03

Supplementary Table 04

Supplementary Table 05

Supplementary Table 06

Supplementary Table 07

Supplementary Table 08

Supplementary Table 09

Supplementary Table 10

Supplementary Table 11

Supplementary Table 12

Supplementary Table 13

Supplementary Table 14

Supplementary Table 15

Supplementary Table 16

## Data Availability

All analyses presented here are based on published data, as cited in “Methods” section. Raw RNA-Seq data for the KCL cohort are available via SRA (https://www.ncbi.nlm.nih.gov/sra) under BioProject ID: PRJNA1040467. Additional information on intermediate data and processing is available from the corresponding author on reasonable request.

## References

[1] McCart Reed AE, Kutasovic JR, Lakhani SR, Simpson PT (2015). Invasive lobular carcinoma of the breast: morphology, biomarkers and ‘omics. Breast Cancer Res.

[2] McCart Reed AE, Kalinowski L, Simpson PT, Lakhani SR (2021). Invasive lobular carcinoma of the breast: the increasing importance of this special subtype. Breast Cancer Res.

[3] Sflomos G, Schipper K, Koorman T, Fitzpatrick A, Oesterreich S, Lee AV (2021). Atlas of lobular breast cancer models: challenges and strategic directions. Cancers.

[4] Wilson N, Ironside A, Diana A, Oikonomidou O (2020). Lobular breast cancer: a review. Front Oncol.

[5] Ciriello G, Gatza ML, Beck AH, Wilkerson MD, Rhie SK, Pastore A (2015). Comprehensive molecular portraits of invasive lobular breast cancer. Cell.

[6] Pestalozzi BC, Zahrieh D, Mallon E, Gusterson BA, Price KN, Gelber RD (2008). Distinct clinical and prognostic features of infiltrating lobular carcinoma of the breast: combined results of 15 International Breast Cancer Study Group clinical trials. J Clin Oncol.

[7] Mathew A, Rajagopal PS, Villgran V, Sandhu GS, Jankowitz RC, Jacob M (2017). Distinct pattern of metastases in patients with invasive lobular carcinoma of the breast. Geburtshilfe Frauenheilkd.

[8] Oesterreich S, Nasrazadani A, Zou J, Carleton N, Onger T, Wright MD (2022). Clinicopathological features and outcomes comparing patients with invasive ductal and lobular breast cancer. J Natl Cancer Inst.

[9] McCart Reed AE, Lal S, Kutasovic JR, Wockner L, Robertson A, de Luca XM (2019). LobSig is a multigene predictor of outcome in invasive lobular carcinoma. NPJ Breast Cancer.

[10] Desmedt C, Zoppoli G, Gundem G, Pruneri G, Larsimont D, Fornili M (2016). Genomic characterization of primary invasive lobular breast cancer. J Clin Oncol.

[11] Sikora MJ, Jacobsen BM, Levine K, Chen J, Davidson NE, Lee AV (2016). WNT4 mediates estrogen receptor signaling and endocrine resistance in invasive lobular carcinoma cell lines. Breast Cancer Res.

[12] Riggins RB, Lan JP, Zhu Y, Klimach U, Zwart A, Cavalli LR (2008). ERRgamma mediates tamoxifen resistance in novel models of invasive lobular breast cancer. Cancer Res.

[13] Luveta J, Parks RM, Heery DM, Cheung KL, Johnston SJ (2020). Invasive lobular breast cancer as a distinct disease: implications for therapeutic strategy. Oncol Ther.

[14] Wishart GC, Azzato EM, Greenberg DC, Rashbass J, Kearins O, Lawrence G (2010). PREDICT: a new UK prognostic model that predicts survival following surgery for invasive breast cancer. Breast Cancer Res.

[15] Paik S, Shak S, Tang G, Kim C, Baker J, Cronin M (2004). A multigene assay to predict recurrence of tamoxifen-treated, node-negative breast cancer. N Engl J Med.

[16] Timbres J, Moss C, Mera A, Haire A, Gillett C, Van Hemelrijck M (2021). Survival outcomes in invasive lobular carcinoma compared to oestrogen receptor-positive invasive ductal carcinoma. Cancers.

[17] Guiu S, Wolfer A, Jacot W, Fumoleau P, Romieu G, Bonnetain F (2014). Invasive lobular breast cancer and its variants: how special are they for systemic therapy decisions?. Crit Rev Oncol Hematol.

[18] Beumer IJ, Persoon M, Witteveen A, Dreezen C, Chin SF, Sammut SJ (2016). Prognostic value of MammaPrint((R)) in invasive lobular breast cancer. Biomark Insights.

[19] Metzger-Filho O, Michiels S, Bertucci F, Catteau A, Salgado R, Galant C (2013). Genomic grade adds prognostic value in invasive lobular carcinoma. Ann Oncol.

[20] Christgen M, Gluz O, Harbeck N, Kates RE, Raap M, Christgen H (2020). Differential impact of prognostic parameters in hormone receptor-positive lobular breast cancer. Cancer.

[21] Filipits M, Rudas M, Jakesz R, Dubsky P, Fitzal F, Singer CF (2011). A new molecular predictor of distant recurrence in ER-positive, HER2-negative breast cancer adds independent information to conventional clinical risk factors. Clin Cancer Res.

[22] Parker JS, Mullins M, Cheang MC, Leung S, Voduc D, Vickery T (2009). Supervised risk predictor of breast cancer based on intrinsic subtypes. J Clin Oncol.

[23] Michaut M, Chin SF, Majewski I, Severson TM, Bismeijer T, de Koning L (2016). Integration of genomic, transcriptomic and proteomic data identifies two biologically distinct subtypes of invasive lobular breast cancer. Sci Rep.

[24] Ben-Hamo R, Jacob Berger A, Gavert N, Miller M, Pines G, Oren R (2020). Predicting and affecting response to cancer therapy based on pathway-level biomarkers. Nat Commun.

[25] Creixell P, Reimand J, Haider S, Wu G, Shibata T, Vazquez M (2015). Pathway and network analysis of cancer genomes. Nat Methods.

[26] Haider S, Yao CQ, Sabine VS, Grzadkowski M, Stimper V, Starmans MHW (2018). Pathway-based subnetworks enable cross-disease biomarker discovery. Nat Commun.

[27] Brueffer C, Vallon-Christersson J, Grabau D, Ehinger A, Hakkinen J, Hegardt C (2018). Clinical value of RNA sequencing-based classifiers for prediction of the five conventional breast cancer biomarkers: a report from the Population-Based Multicenter Sweden Cancerome Analysis Network-Breast Initiative. JCO Precis Oncol.

[28] Brueffer C, Gladchuk S, Winter C, Vallon-Christersson J, Hegardt C, Hakkinen J (2020). The mutational landscape of the SCAN-B real-world primary breast cancer transcriptome. EMBO Mol Med.

[29] Schemper M, Smith TL (1996). A note on quantifying follow-up in studies of failure time. Control Clin Trials.

[30] Ritchie ME, Phipson B, Wu D, Hu Y, Law CW, Shi W (2015). limma powers differential expression analyses for RNA-sequencing and microarray studies. Nucleic Acids Res.

[31] Haider S, McIntyre A, van Stiphout RGPM, Winchester LM, Wigfield S, Harris AL (2016). Genomic alterations underlie a pan-cancer metabolic shift associated with tumour hypoxia. Genome Biol.

[32] Subramanian A, Tamayo P, Mootha VK, Mukherjee S, Ebert BL, Gillette MA (2005). Gene set enrichment analysis: a knowledge-based approach for interpreting genome-wide expression profiles. Proc Natl Acad Sci USA.

[33] Vijaymeena MK, Kavitha K (2016). A survey on similarity measures in text mining. Mach Learn Appl.

[34] Rousseeuw PJ (1987). Silhouettes: a graphical aid to the interpretation and validation of cluster analysis. J Comput Appl Math.

[35] Maechler M, Rousseeuw P, Struyf A, Hubert M, Hornik K. cluster: cluster analysis basics and extensions. R package version 2.0.3. 2015.

[36] Gendoo DM, Ratanasirigulchai N, Schroder MS, Pare L, Parker JS, Prat A (2016). Genefu: an R/Bioconductor package for computation of gene expression-based signatures in breast cancer. Bioinformatics.

[37] Curtis C, Shah SP, Chin SF, Turashvili G, Rueda OM, Dunning MJ (2012). The genomic and transcriptomic architecture of 2,000 breast tumours reveals novel subgroups. Nature.

[38] Lips EH, Mukhtar RA, Yau C, de Ronde JJ, Livasy C, Carey LA (2012). Lobular histology and response to neoadjuvant chemotherapy in invasive breast cancer. Breast Cancer Res Treat.

[39] Thomas M, Kelly ED, Abraham J, Kruse M (2019). Invasive lobular breast cancer: a review of pathogenesis, diagnosis, management, and future directions of early stage disease. Semin Oncol.

[40] Guedj M, Marisa L, de Reynies A, Orsetti B, Schiappa R, Bibeau F (2012). A refined molecular taxonomy of breast cancer. Oncogene.

[41] Butler D, Rosa M (2013). Pleomorphic lobular carcinoma of the breast: a morphologically and clinically distinct variant of lobular carcinoma. Arch Pathol Lab Med.

[42] Narendra S, Jenkins SM, Khoor A, Nassar A (2015). Clinical outcome in pleomorphic lobular carcinoma: a case-control study with comparison to classic invasive lobular carcinoma. Ann Diagn Pathol.

[43] van ‘t Veer LJ, Dai H, van de Vijver MJ, He YD, Hart AA, Mao M (2002). Gene expression profiling predicts clinical outcome of breast cancer. Nature.

[44] Venet D, Dumont JE, Detours V (2011). Most random gene expression signatures are significantly associated with breast cancer outcome. PLoS Comput Biol.

[45] Haider S, Wang J, Nagano A, Desai A, Arumugam P, Dumartin L (2014). A multi-gene signature predicts outcome in patients with pancreatic ductal adenocarcinoma. Genome Med.

[46] Boutros PC, Lau SK, Pintilie M, Liu N, Shepherd FA, Der SD (2009). Prognostic gene signatures for non-small-cell lung cancer. Proc Natl Acad Sci USA.

[47] Bajrami I, Marlow R, van de Ven M, Brough R, Pemberton HN, Frankum J (2018). E-Cadherin/ROS1 inhibitor synthetic lethality in breast cancer. Cancer Discov.

[48] Metzger Filho O, Giobbie-Hurder A, Mallon E, Gusterson B, Viale G, Winer EP (2015). Relative effectiveness of letrozole compared with tamoxifen for patients with lobular carcinoma in the BIG 1-98 trial. J Clin Oncol.

[49] Lin A, Sheltzer JM (2020). Discovering and validating cancer genetic dependencies: approaches and pitfalls. Nat Rev Genet.

[50] Barretina J, Caponigro G, Stransky N, Venkatesan K, Margolin AA, Kim S (2019). Addendum: the cancer cell line encyclopedia enables predictive modelling of anticancer drug sensitivity. Nature.

[51] Slamon DJ, Clark GM, Wong SG, Levin WJ, Ullrich A, McGuire WL (1987). Human breast cancer: correlation of relapse and survival with amplification of the HER-2/neu oncogene. Science.

[52] Li S, Shen Y, Wang M, Yang J, Lv M, Li P (2017). Loss of PTEN expression in breast cancer: association with clinicopathological characteristics and prognosis. Oncotarget.

[53] Oh DY, Bang YJ (2020). HER2-targeted therapies—a role beyond breast cancer. Nat Rev Clin Oncol.

[54] Meyers RM, Bryan JG, McFarland JM, Weir BA, Sizemore AE, Xu H (2017). Computational correction of copy number effect improves specificity of CRISPR-Cas9 essentiality screens in cancer cells. Nat Genet.

[55] Laenkholm AV, Jensen MB, Eriksen JO, Roslind A, Buckingham W, Ferree S (2020). Population-based study of Prosigna-PAM50 and outcome among postmenopausal women with estrogen receptor-positive and HER2-negative operable invasive lobular or ductal breast cancer. Clin Breast Cancer.

[56] Shimada K, Bachman JA, Muhlich JL, Mitchison TJ (2021). shinyDepMap, a tool to identify targetable cancer genes and their functional connections from Cancer Dependency Map data. Elife.

[57] Williamson CT, Miller R, Pemberton HN, Jones SE, Campbell J, Konde A (2016). ATR inhibitors as a synthetic lethal therapy for tumours deficient in ARID1A. Nat Commun.

[58] Lecona E, Fernandez-Capetillo O (2018). Targeting ATR in cancer. Nat Rev Cancer.

