# Supplementary material for: Pathway-based signatures predict patient outcome, chemotherapy benefit and synthetic lethal dependencies in invasive lobular breast cancer
Source: Br J Cancer. 2024 Apr 10;130(11):1828–40. doi: 10.1038/s41416-024-02679-7 (PMC11130209; doi:10.1038/s41416-024-02679-7)
Supplement: Supplementary file 1 — Supplementary Materials [file 41416_2024_2679_MOESM1_ESM.pdf]

Pathway-based signatures predict patient outcome, chemotherapy benefit and synthetic lethal dependencies in invasive lobular breast cancer

John Alexander[1], Koen Schipper[1], Sarah Nash[1,2], Rachel Brough[1,3], Harriet Kemp[1], Jacopo Iacovacci[1], Clare Isacke[1], Rachael Natrajan[1], Elinor Sawyer[2], Christopher J Lord[1,3], Syed Haider[1,*]

Affiliations:

[1] The Breast Cancer Now Toby Robins Research Centre, The Institute of Cancer Research, London, SW3 6JB, UK

[2] Breast Cancer Genetics, King's College London, London SE1 9RT, UK

[3] CRUK Gene Function Laboratory, The Institute of Cancer Research, London, SW3 6JB, UK

*Correspondence: Syed.Haider@icr.ac.uk

Supplementary Figures and Legends

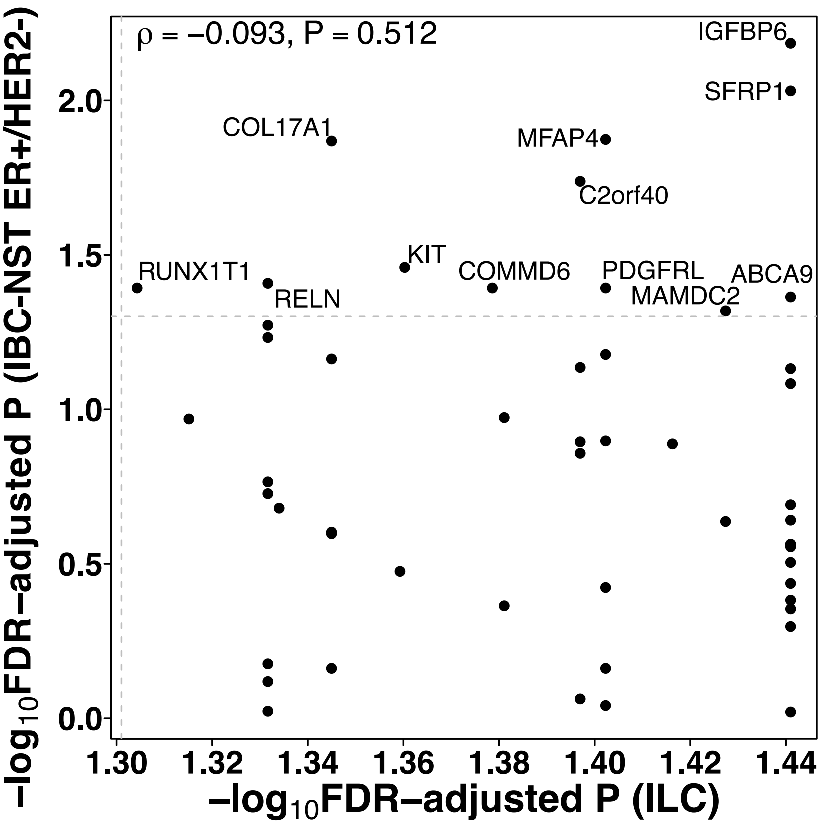

Supplementary Figure 1

Scatter plot showing -$\log_{10}$ FDR-adjusted P values (Cox proportional hazards model) between Metabric ILC (n=147) and ER+/HER2- IBC-NST (n=1046) samples. Each point represents a gene that was identified as a candidate prognostic gene in the Metabric ILC samples. Genes that were prognostic (FDR-adjusted P < 0.05) in both ILC and ER+/HER2- IBC-NST and followed the same direction of coefficient ($\log_2$(HR)) are labelled.

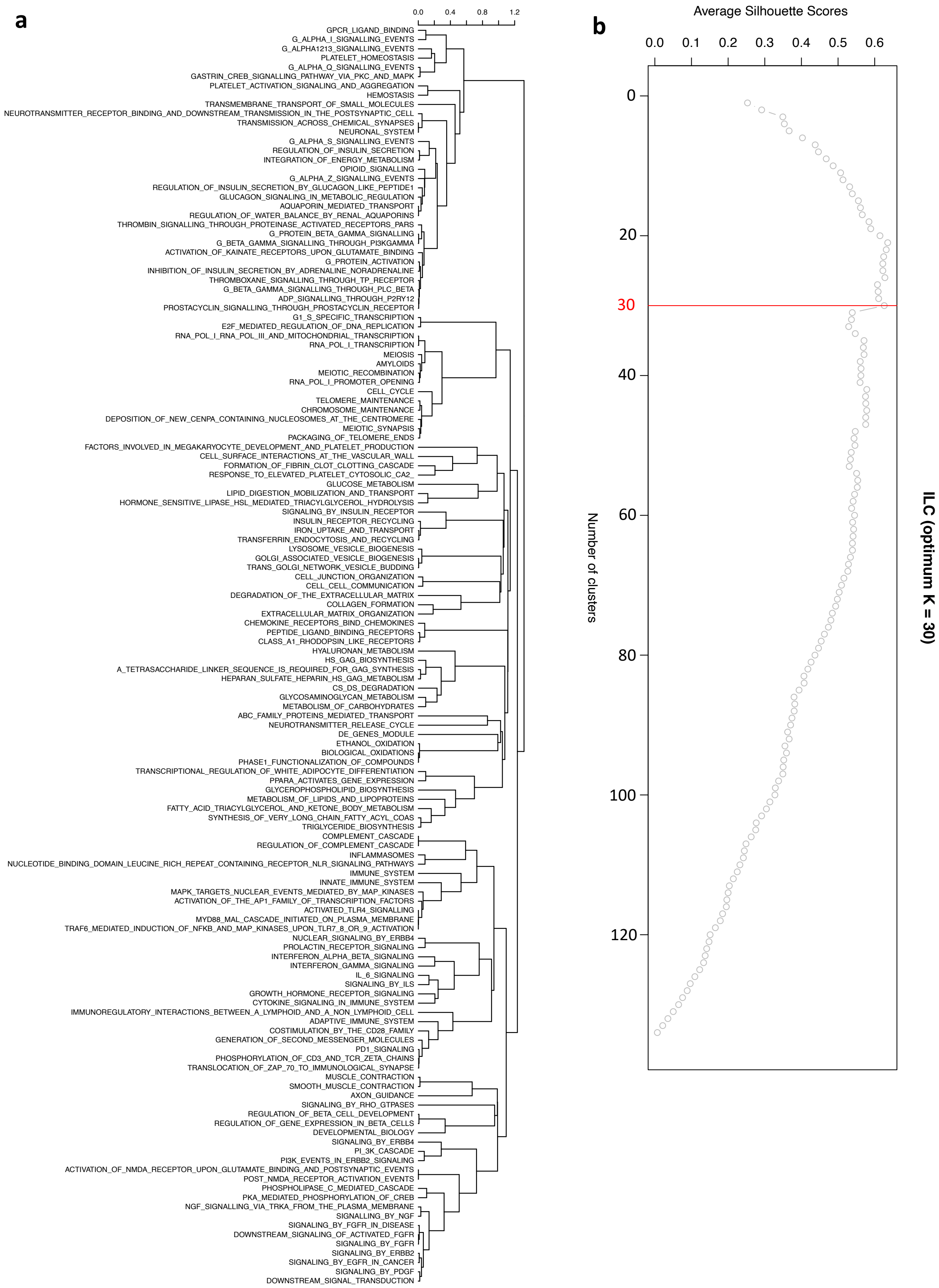

Supplementary Figure 2

a) Hierarchical clustering of the overlap coefficients estimated between 135 significantly enriched pathways identified from the over-representation analysis on 1,398 ILC

dysregulated genes, along with an additional module comprised of significantly differentially expressed/variable genes that did not map to any of the significantly enriched pathways.

b) Graph of average Silhouette values for k = 2 to 135. Red line shows the optimal number of overlapping clusters (k=30) with high Silhouette score while maintaining largest possible number of clusters.

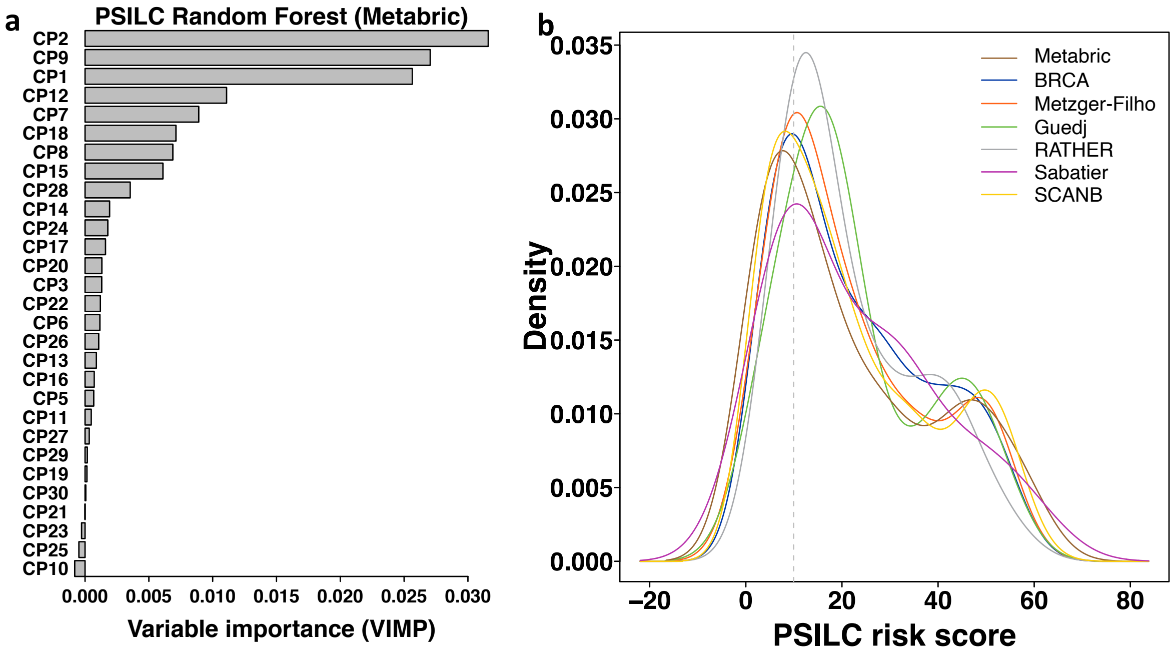

Supplementary Figure 3

a) Bar plot of variable importance scores of 29 CPs from the PSILC multivariable prognostic model using the random forest algorithm. CP04 was not included in the analysis because none of its genes were prognostic in the discovery set (Wald P < 0.05).

b) Density plot of PSILC risk scores in discovery (Metabric) and validation cohorts

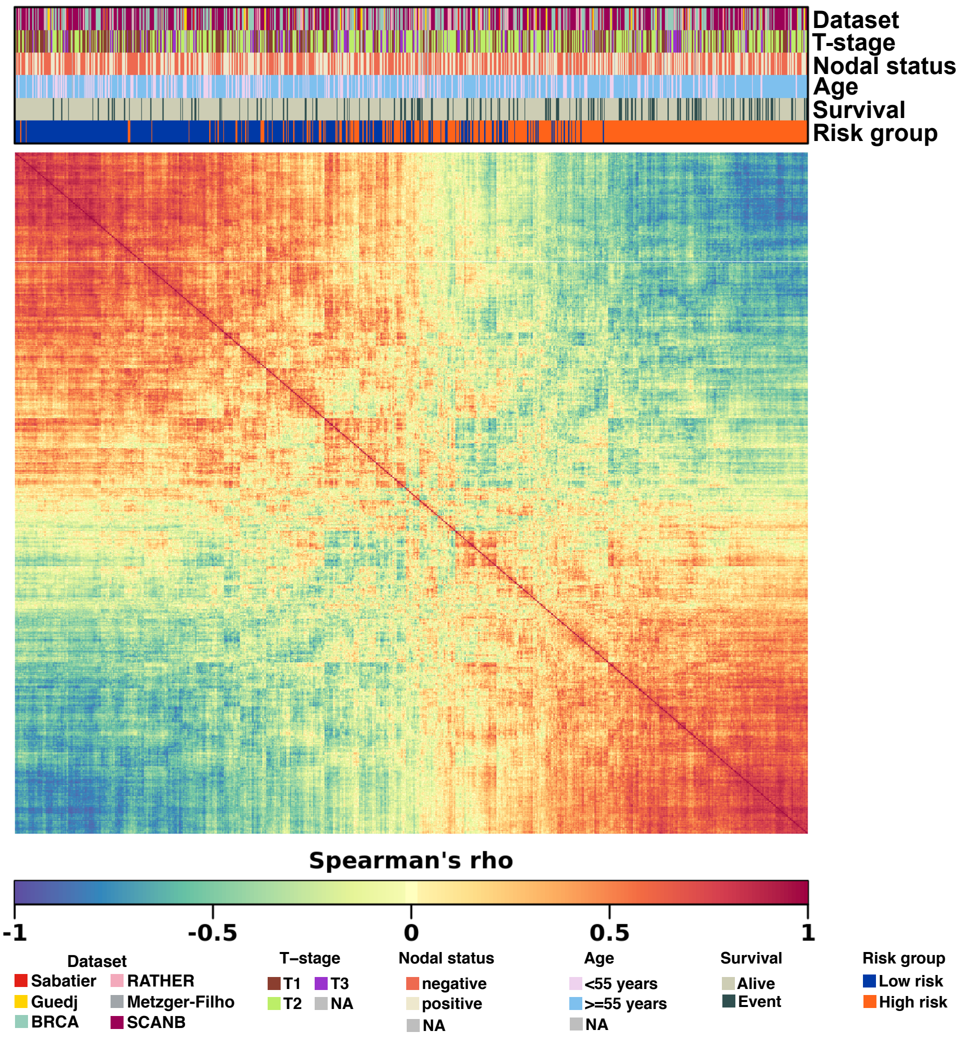

Supplementary Figure 4

Heatmap of inter-patient correlation (Spearman's rho) using risk scores from 29 CPs in the validation cohorts. Each column and row represent a patient's correlation with all other patients. Column covariates show patient classifications based on dataset, tumour stage (T-stage), nodal status, age, survival event and predicted risk group.

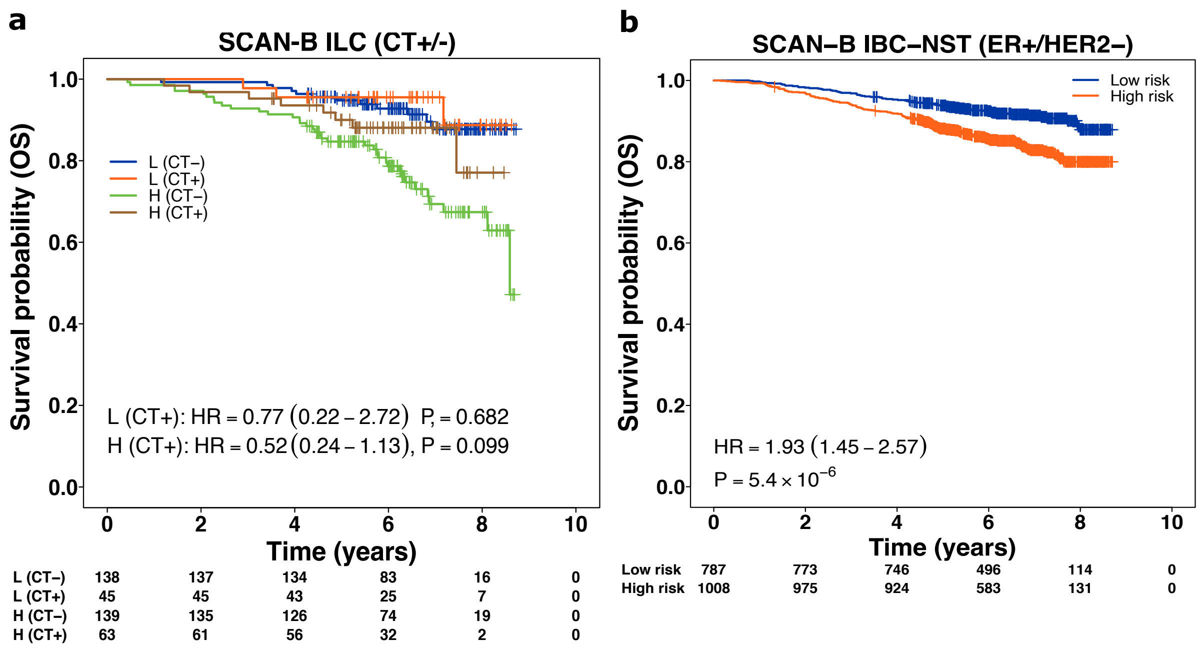

Supplementary Figure 5

a) Kaplan-Meier survival curves in the SCAN-B ILC dataset, stratified by high and low-risk group patients in chemotherapy treated (CT+) and chemotherapy naïve (CT-) subgroups.

b) Kaplan-Meier survival curves in the SCAN-B IBC-NST ER+/HER2- dataset.

Hazard ratios (estimated using the Cox proportional hazards model) and P values of Wald-test are reported.

Supplementary Table Legends

Supplementary Table 1
Summary of clinical cohorts used in this study.

Supplementary Table 2
Differential gene expression analysis using LIMMA between ILC and normal breast samples.

Supplementary Table 3
Differential gene variation analysis using iDOS (f-test) between ILC and normal breast samples.

Supplementary Table 4
Prognostic assessment of significantly dysregulated genes (Supplementary Tables 3 and 4). A Cox proportional hazards model was fitted to continuous mRNA abundance profiles. Positive beta indicates high mRNA abundance associated with poor outcome while negative beta indicates high mRNA abundance associated with good outcome.

Supplementary Table 5
List of significantly enriched pathways (FDR-adjusted $P < 0.1$) using over-representation analysis. P value is estimated using Fisher's exact test.

Supplementary Table 6
Clustering of pathways based on shared genes. Column: 'Cluster (CP)' indicates clustered group following merging of overlapping pathways/sub-pathways into a single cluster of pathways (CP) guided by the Silhouette coefficient. Subsequent columns indicate three top level classes of pathways for a given CP.

Supplementary Table 7
Prognostic assessment of CPs using Cox proportional hazards model.

Supplementary Table 8
Prognostic assessment of CPs in chemotherapy naïve subgroup (SCAN-B CT- cohort).

Supplementary Table 9
Prognostic assessment of CPs in chemotherapy treated subgroup (SCAN-B CT+ cohort).

Supplementary Table 10
Test of interaction between chemotherapy and risk groups in the SCAN-B cohort using a Cox proportional hazards model.

Supplementary Table 11
Estimated parameters of the multivariable PSILC two risk group and three risk group classifications.

Supplementary Table 12
Estimated parameters of the multivariable PSILC two risk group and three risk group classifications in combined validation cohort when adjusted for age, T-stage, nodal status and tumour purity. Data is shown for samples where clinical covariates were available.

Supplementary Table 13
Estimated parameters of the multivariable PSILC two risk group in the pleomorphic ILC cohort (KCL).

Supplementary Table 14
Comparison of CRISPR gene effect profiles between PSILC high and low group of ILC/ILC-like cell lines. One-sided two sample Welch's t-test was used for statistical comparisons.

Supplementary Table 15
Comparison of CRISPR gene effect profiles between PSILC high (ERBB2-WT) and low groups of ILC/ILC-like cell lines. One-sided two sample Welch's t-test was used for statistical comparisons.

Supplementary Table 16
Pathway over-representation analysis of candidate synthetic lethal genes identified from the CRISPR-Cas9 gene effect analysis.
